# Characterization of Fitness Profiles in Youth Soccer Players in Response to Playing Roles Through Principal Component Analysis

**DOI:** 10.3390/jfmk10010040

**Published:** 2025-01-21

**Authors:** Boryi A. Becerra Patiño, Aura D. Montenegro Bonilla, Juan D. Paucar-Uribe, Diego A. Rada-Perdigón, Jorge Olivares-Arancibia, Rodrigo Yáñez-Sepúlveda, José Francisco López-Gil, José Pino-Ortega

**Affiliations:** 1 Faculty of Physical Education, National Pedagogical University, Valmaria Cl. 183 # 5199, Bogotá 480100, Colombia; admontenegrob@upn.edu.co (A.D.M.B.); jdpaucaru@upn.edu.co (J.D.P.-U.); daradap@upn.edu.co (D.A.R.-P.); 2 Management and Pedagogy of Physical Activity and Sport (GPAFD), Faculty of Physical Education, National Pedagogical University, Valmaria Cl. 183 # 5199, Bogotá 480100, Colombia; 3 AFySE Group, Research in Physical Activity and School Health, School of Physical Education, Faculty of Education, Universidad de las Américas, Santiago 7500000, Chile; jolivares@udla.cl; 4 Faculty Education and Social Sciences, Universidad Andres Bello, Viña del Mar 2520000, Chile; rodrigo.yanez.s@unab.cl; 5 One Health Research Group, Universidad de Las Américas, Quito 170124, Ecuador; 6 Faculty of Sport Science, University of Murcia, 30100 Murcia, Spain; josepinoortega@um.es

**Keywords:** team sports, assessment, physical fitness, playing position, role of play

## Abstract

**Background/Objectives:** Physical fitness in youth soccer impacts individual and team performance through the specific demands that must be met on the field. Therefore, this study aimed to characterize and identify youth soccer players with regard to the roles they play on the field. **Material and Methods:** A cross-sectional study was designed to characterize and identify the physical fitness levels of youth soccer players using previously validated measurement tests. A total of 36 players were evaluated (15 defenders and 24 attackers) using various physical fitness tests: Squat Jump (SJ), Countermovement Jump (CMJ), Single-leg Countermovement Jumps (SLCMJs), COD-Timer 5-0-5, Speed (5, 10, 15, and 20 m), Yo-Yo Intermittent Recovery Test Level I (YYIR1), and Running-Based Anaerobic Sprint Test (RAST). The data were confirmed using the Shapiro–Wilk test. Effect sizes were obtained using the Rank-Biserial coefficient, and, to identify the profiles of attackers and defenders, principal component analysis (PCA) was employed. **Results:** For the strength variables, attackers obtained better results than defenders in the variable flight time in the SJ (*p* = 0.03; R-b = −0.33) and contact time (%) in the SLCMJ test (*p* = 0.04; R-b = −0.33). Meanwhile, defenders achieved better results than attackers in the SLCMJ test for the variable flight time (%) (*p* = 0.01; R-b = 0.33) and breaking angle (A°) in the Nordic Hamstring (*p* = 0.01; R-b = 0.33). The results showed differences according to the players’ roles. Three principal components were identified for both attackers and defenders. The PC1 for attackers considered variables of strength, asymmetry, change of direction, and power. PC2 only considered strength and power variables. PC3 considered variables of strength, speed, endurance, and power. For defenders, PC1 considered strength, asymmetry, and power. PC2 analyzed variables of strength, asymmetry, change of direction and power. Finally, PC3 only grouped speed variables. **Conclusions:** Although youth soccer positions involve offensive and defensive roles, this study reveals differences in certain physical fitness variables. Therefore, it is necessary to tailor training tasks according to the specificity of the playing position, in line with the systems of play used and the predominance of the role that players occupy, whether in defense or attack.

## 1. Introduction

Youth soccer performance is multifaceted, and biological maturation and physical fitness as markers of physical development in the analysis of players depend on the environment to which the players are exposed [1]. Thus, physical development in youth may condition sport performance in adulthood [2]. This has been reported in different studies, where the specific characteristics of a game have been studied, including strength and power [3], speed [4], and the ability to repeat explosive efforts [5], which allows the player to adapt to the needs of the competition [6].

Soccer players perform a variety of movements during training and competitions, where they must be able to respond to the physical demands of the game [7], taking into account the sports technique and the specific characteristics of the sport to perform repeated or variable actions [8]. These actions enable players to express superiority in competition [3] and improve decision making on the field [9].

For this reason, success in team sports is associated with the development of psychological, physiological, motor, and physical abilities that impact sports performance [10,11]. These advances are established in youth stages, where conditions are developed to achieve elite fitness [12], based on specific soccer learning, and are associated with real movements in competition [13].

Movements preceding a specific sport action are theoretically known as performance enhancement, especially through jumping actions [14], for which exercises must be prescribed according to the principle of similarity in order for this effect to be effective in practice [15]. The basis lies in activating the specific muscles used in sports movement [16] through the metabolic and mechanical changes that occur in the sport [17], specifically in response to the physical demands in youth soccer [18,19]. This physical demand is characterized by the expression of various abilities related to strength, speed, and power [20,21].

Some of the most studied exercises in lower body strength training include the Squat Jump (SJ) [22], Countermovement Jump (CMJ) [23], and Countermovement with Arms (Abalakov) [24,25]. Strength training is one of the most effective methods for the comprehensive development of young athletes [26], as is well documented in several meta-analyses [27]. Consequently, one of the key variables in the physical fitness of players has been the analysis of explosive strength performance [28] through plyometric exercises, which are based on constant cycles of stretch shortening. In these cycles, the stretching phase (eccentric phase) is followed by a shortening phase (concentric phase), executed in the shortest possible time [29,30], with the second phase being co-dependent on the first [29,31].

Strength has also proven to be useful in improving other abilities [32], as well as lower body asymmetries, which can help determine the risk of sports injuries [33] or performance decline [34]. However, scientific evidence has not been clear in yielding reliable conclusions on asymmetries [35], as demonstrated by Bishop et al. [36] in a systematic analysis, where imbalances were seen to have negative effects. On the other hand, the study by Coratella et al. [37] concluded that maximal torque asymmetry between the quadriceps and hamstrings correlates with COD and sprinting ability, while it does not correlate with the SJ and CMJ. Difficulties in understanding them persist, making it necessary to continue researching asymmetries in different sports contexts.

Other risk factors include non-contact injuries to the hamstring muscles [38,39], as these muscles are predominantly bi-articular, linking the hip and knee and playing a role during walking and running [40]. Nordic training is one of the most notable indicators for injury prevention and reducing injury risk [41]. Despite this, there are still theoretical gaps that need further investigation [42]. Moreover, other important physical abilities for soccer players include acceleration and deceleration [43], such as sprints [44] and changes of direction [45]. Players can cover up to 8448 m in activities with these characteristics [46], often influenced by external stimuli such as the ball, opponents, and teammates [45]. Reliable and validated tests for youth soccer players have been confirmed [47].

Respiratory capacity has been assessed using various instruments [9,48]; however, it is relevant to consider the physical demands of soccer [49], which requires high-intensity intermittent actions through both aerobic and anaerobic pathways [50]. Therefore, in recent years, the Yo-Yo Intermittent Recovery Test Level I (YYIR1) and the Running-based Anaerobic Sprint Test (RAST) have been implemented [51]. Sprints with these cardiorespiratory demands tend to decrease throughout the match, which is associated with the fatigue index [52]. Understanding this suggests that optimal levels could minimize fatigue during power actions [53], and this becomes more complex since these actions are linked to good motor development and musculoskeletal synchronization for sports movements [54].

It is relevant to evaluate neuromuscular factors, which have been associated with strength and speed capacity [55]. These factors contribute to improvements in high-intensity intermittent cardiorespiratory capacity [56], jump height [57,58], acceleration and deceleration exercises [59], hamstring strength [60], and lower limb asymmetries [33,34]. However, these actions have been analyzed on the basis of establishing significant differences between training and competition variables, as well as between different groups of physical and tactical variables [61]. Thus, to reduce the large number of variables analyzed in the different studies, principal component analysis (PCA) is a multivariate statistical technique that has been widely used in different studies to establish patterns in data sets [62]. The dimensionality of the data allows for the extraction of principal components (PCs) that capture the greatest variance of the data analyzed; therefore, a PCA provides valuable information between different groups of variables to create profiles [63]. Thus, in collective sports, PCA has been widely used for analysis between playing positions and important indicators for sport performance [63,64,65,66].

Therefore, the objectives of this study were (i) to identify key performance indicators in youth soccer players and (ii) to determine the existence of fitness profiles of youth soccer players regarding the roles they play on the field.

## 2. Materials and Methods

### 2.1. Design and Procedures

A cross-sectional study [67] was designed to characterize and identify the physical fitness levels of youth soccer players using previously validated measurement tests. The field tests were conducted over several sessions, always at the same time (4:00 to 6:00 p.m.), under similar environmental conditions (average temperature: 13.1 °C; humidity: 77–83%), and without fasting conditions. Before starting, the players underwent a standardized warm-up protocol. Running exercises from the FIFA 11+ manual were performed continuously for 40 s to 1 min. The first exercise involved running in a straight line within a designated space at moderate intensity. Then, straight-line running was performed, with players moving their hips outward and inward with each step to activate the lateral and internal hip muscles and improve lateral mobility. Next, players ran in circles around a teammate, alternating directions. Afterward, they performed straight-line running, jumping upon contact with a teammate at a certain distance. Finally, fast straight-line sprints were conducted, alternating forward and backward directions.

### 2.2. Participants

This study was conducted with 36 soccer players. The players were selected as defenders and attackers according to the role they played the most and according to the game model of the club being evaluated. Thus, the players selected as defenders were goalkeepers, central defenders, lateral defenders, and midfielders, while the attackers were lateral midfielders, attacking midfielders, and forwards. Thus, the aim was to determine the differences that may exist depending on the predominant playing role in the game in order to continue providing scientific evidence that contributes to the understanding of children’s and youth soccer.

The main characteristics of the sample are provided below (Table 1). Based on the described experimental design, with a type I error of 5% and a nominal power of 80%, the sample size was determined using Cohen’s d statistic, referencing the detection of strong differences between groups (defenders and attackers). Given the sample sizes under the given conditions, the groups consisted of 18 and 19 athletes (power: 0.801). Based on the study’s participation criteria, the final sample sizes were 15 and 21, with a power score close to the nominal value (power: 0.779).

All players met the following inclusion criteria: (i) absence of musculoskeletal injury or health issues in the two months before the assessments, (ii) a minimum of two years of training experience, (iii) completion of all physical fitness tests, (iv) training at least 3 times a week plus one competition on the weekend, and (v) being within the age range of 13–14 years. Conversely, the exclusion criteria were (i) presenting pathological conditions that prevented physical and cognitive effort during the tests, (ii) having been with the club for less than three months, (iii) returning from an injury or being in a rehabilitation period, and (iv) training without competing. Thus, all the players evaluated were healthy enough to be able to perform each of the tests considered for this study. Finally, this study was conducted during the first part of the season period, with a minimum reference of 48 h, two days before and two days after the competition, to carry out the assessments.

### 2.3. Equipment

For body mass evaluation, the OMROM scale model HBF-514C (Kyoto, Japan) was used, with an accuracy of 0.1 cm. Height was measured using a portable stadiometer (Seca, 213) (Hamburg, Germany). For the physical fitness assessments, the following tests were performed: Countermovement Jump (CMJ), Squat Jump (SJ) [68], Nordic Hamstring, Single-Leg Countermovement Jumps (SLCMJs) [69], COD-Timer 5-0-5 [70], and speed using the Runmatic app [71]. The electronic device used was an iPhone 14 model 2023 (Cupertino, CA, USA). For the research testing phase, materials were used according to the evaluated structure. For the RAST [72], various equipment was used, such as cones to mark the 35 m distance covered in each run, a measuring tape for distance measurement, and two stopwatches to time arrivals on each side. For the YYIR1 test [73], cones were used to mark the 20 m zone, along with a measuring tape for measurement and a speaker for start cues.

### 2.4. On-Court Physical Fitness Tests and Registered Variables

Various validated physical fitness tests were applied to characterize and identify the physical performance level of youth soccer players in relation to their role on the field. On the first day, strength tests were conducted, measured through different jumps (CMJ, SJ, SLCMJs). On the second day, Nordic Hamstring and RASTs were performed. The third day included the COD-Timer 5-0-5 and speed tests, and, finally, on the fourth day, the YYIR1 test was evaluated. Each day’s assessments were spaced 48 h apart. For the jump tests, the recording device was positioned two meters away from the athlete’s jump area. Below is a description of the tests and variables obtained, following the order in which they were conducted during the study, with each test separated by two minutes of passive recovery [74].

#### 2.4.1. CMJ

The Countermovement Jump (CMJ) allows the participant to freely flex their legs and react by pushing off with momentum; the CMJ measures the ability to generate force over a longer time compared to the SJ. Before performing each CMJ, participants were instructed to “jump as high and fast as possible” with their hands fixed on their hips [75].

#### 2.4.2. SJ

The Squat Jump (SJ) protocol involves jumping as high as possible with hands on the hips, starting from a position with the tibiofemoral joint at 90 degrees. The SJ measures the quality of the take-off, the non-plyometric vertical jump, and the ability to develop force quickly, i.e., explosiveness [76].

#### 2.4.3. SLCMJs

This test is a tool to evaluate and measure strength and performance differences between muscles. It also helps identify muscle imbalances and assess variables such as push-off distance, contact time, contact asymmetry, contact time, and flight time with both the left and right legs. In this test, balance and strength in both legs are particularly relevant [69].

#### 2.4.4. Hamstring Strength

This test is used to evaluate the strength and endurance of the hamstring muscles, particularly the hamstrings, which include the biceps femoris, semitendinosus, and semimembranosus muscles located at the back of the thigh. The investigator ensured the correct implementation of the hamstring strength curl (NHCBP). The break point of the NHCBP movement was determined by motion analysis. In the analysis, an iPhone 14 camera was set at 240 fps and placed approximately 3 m from the right side of the participants at a height of approximately 0.9 m. Digitization determined the angle from the knee to the ground when the athlete lost balance and stopped moving in the NHCBP as the break point [77]. The test is performed using the My Jump Lab 2 application, analyzing torque data and breaking angle [78].

#### 2.4.5. COD-Timer 5-0-5

The 5-0-5 COD test is a well-established protocol for measuring change of direction performance and involves a high-intensity cut, often performed in competitions. Changes of direction (CODS) were evaluated using the COD-Timer app for iPhone (version 2), which provides total time measurement (r = 0.964; confidence interval (CI) 95% = 0.95–1.00; standard error of the estimate = 0.03 s; *p* < 0.001) [70].

#### 2.4.6. Speed (5, 10, 15, and 20 m)

This 5, 10, 15, and 20 m test is a physical assessment used in soccer to measure a player’s speed over specific distances. At 5 m, it measures acceleration speed over a short distance. At 10 m, it evaluates acceleration speed and the ability to maintain that speed over a slightly longer distance. At 15 m, it measures speed over a medium distance, and at 20 m, it assesses speed over a longer distance, providing information about the player’s maximum speed in a longer sprint [71].

#### 2.4.7. RAST

The RAST is a test that measures a runner’s anaerobic capacity. This test is conducted on a track and consists of running four maximum sprints of 35 m, with a 10 s rest between each sprint. The time for each sprint can be measured with a photo timer and stopwatches and is recorded to calculate the distance covered and speed. The final result of the test is the total time taken to complete the four sprints, which is used to estimate the runner’s anaerobic capacity [72].

#### 2.4.8. YYIR1

Also known as the Yo-Yo Intermittent Recovery Test Level 1 (YYIR1), this fitness test is used to measure an individual’s ability to perform high-intensity intermittent exercise. It is a useful tool for assessing intermittent endurance capacity in young soccer players and is reliable and stable for evaluation [73]. The YYIR1 consists of multiple periods of running and resting to cover the greatest possible distance before failing to keep up with the audio file’s pace. The distance covered by the athlete at each level of the test is measured and recorded and is used to determine the athlete’s score [73]. The test is divided into several levels, each with increasing intensity and duration. Each level consists of a running period followed by an active (walking) or passive (standing) recovery period for a specified time. Athletes must keep pace with the audio file that indicates the end of the recovery period and the start of the next running period. The test ends when an athlete fails to reach the cone line in the required time or fails to keep pace with the audio file twice in a row. The total distance covered during the test is used to calculate the athlete’s score. The YYIR1 test has proven to be a reliable and valid test for assessing aerobic capacity and endurance across a wide range of sports and populations. It is widely used in sports such as soccer, basketball, handball, rugby, and volleyball, among others [73].

### 2.5. Procedures

Before initiating all the procedures, this study complied with regulations, following the ethical guidelines of the Declaration of Helsinki and under the principles established by Resolution 8430 of the Ministry of Health of Colombia, declaring the study to be of low risk according to Colombian regulations based on standards and guidelines for research involving non-invasive procedures. Finally, this study received approval from the ethics committee of the National Pedagogical University (340ETIC-2024). The club was then contacted to inform them about the study proposal. The club managers, technical staff, and guardians of the players signed the informed consent. After this, the date for the test was agreed upon with the club and the coaches of the categories. Before the tests, the researchers conducted several familiarization sessions to familiarize the athletes with the tests and the high monitoring involved, reducing the chances of error during the evaluation. Players were summoned 15 min before the evaluation to organize them into groups of three for the physical assessment. At the end of the physical tests, the research team downloaded the data onto a laptop and imported them to obtain the variables. The data were exported to an Excel spreadsheet, and a database was created in Excel before being entered into the statistical package for subsequent analysis.

### 2.6. Statistical Analysis

The results of the various tests on soccer players according to their positions are reported as mean and standard deviation (SD). The normality and homoscedasticity of the data were confirmed using the Shapiro–Wilk test, with the results showing that the data did not follow a normal distribution. The differences between the various tests between attackers and defenders were analyzed using the non-parametric Wilcoxon test. The following *p*-values were established (* *p* < 0.05). Effect sizes were obtained using the Rank-Biserial coefficient. The interpretation of r_b is as follows: r_b close to 0 indicates that there is no significant difference between the groups; positive r_b indicates that attackers tend to have higher ranks compared to defenders; and negative r_b indicates that attackers tend to have lower ranks compared to defenders. Then, to identify the profile of each test in attackers and defenders, principal component analysis (PCA) was used [79]. The variables were scaled and centered (Z-score). To define the statistical parameter of the PCA, the determinant of the Kendall correlation matrix was used. In both cases, a value close to 0 was obtained, indicating high multicollinearity and suggesting that the variables have significant linear relationships with most of the variability in the data concentrated in the first three dimensions. Eigenvalues > 1 were considered for the extraction of principal components. An orthogonal Varimax rotation method was performed to identify the high correlation of the components and ensure that each principal component provided different information. A threshold of 0.5 was maintained for each PC loading for interpretation. The values assigned to each observation of all athletes and the 57 variables were appended. All analyses were performed using the software RStudio version 4.1.0 (RStudio, INC, Boston, MA, USA, 2016).

## 3. Results

### 3.1. Differences Related to the Roles Played in the Physical Fitness Level of Young Soccer Players

The differences in the physical fitness level of young soccer players between defenders and attackers are presented in Table 2. For the strength variables, attackers obtained better results than defenders in the variable flight time in the SJ (*p* = 0.03; R-b = −0.33) and contact time (%) in the SLCMJ test (*p* = 0.04; R-b = −0.33). Meanwhile, defenders achieved better results than attackers in the SLCMJ test for the variable flight time (%) (*p* = 0.01; R-b = 0.33) and breaking angle (A°) in the Nordic Hamstring test (*p* = 0.01; R-b = 0.33).

No differences were reported for any of the distances (5, 10, 15, and 20 m) in the speed test. However, for the COD-Timer 5-0-5 test, significant differences were found for the time (s) variable (*p* = 0.01; R-b: 0.33), with attackers being faster than defenders (Table 3).

No differences were reported for the total distance covered or for Vo2max in the YYIR1 test. Meanwhile, significant differences were found for the RAST in T1 (*p* = 0.01; R-b: 0.39), Power T1 (*p* = 0.01; R-b: −0.33), T2 (*p* = 0.03; R-b: 0.33), Power T2 (*p* = 0.01; R-b: −0.33), and maximum power (*p* = 0.01; R-b: −0.33), with attackers being more powerful than defenders (Table 4).

### 3.2. Physical Fitness Profile According to the Roles Played by Young Soccer Players

**Figure 1:**
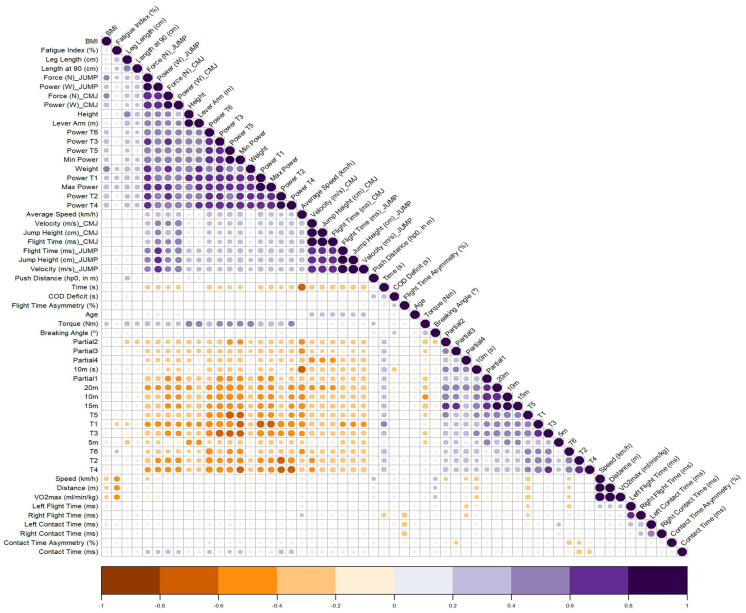
Kendall relationship matrix. Note: in the Kendall correlation, when the circles are larger, they indicate a higher correlation, the color is also adjusted, that is, if the circle is dark, it will be larger and a positive correlation, if we have small circles, we see that they are lighter, when we have orange tones, the pattern is the same, but the correlations are negative.

The Kendall correlation matrix reveals how the variables have positive or negative relationships. Thus, it is evident in Figure 1 that the maximum power variable from the RAST has a positive relationship (0–1) with the strength and power variables from the SJs and CMJs and the power achieved in the first sprint of the RAST. Conversely, the 10 m speed test shows a negative relationship (−1–0) with the average speed (km/h) in the COD-Timer 5-0-5 test.

**Figure 2:**
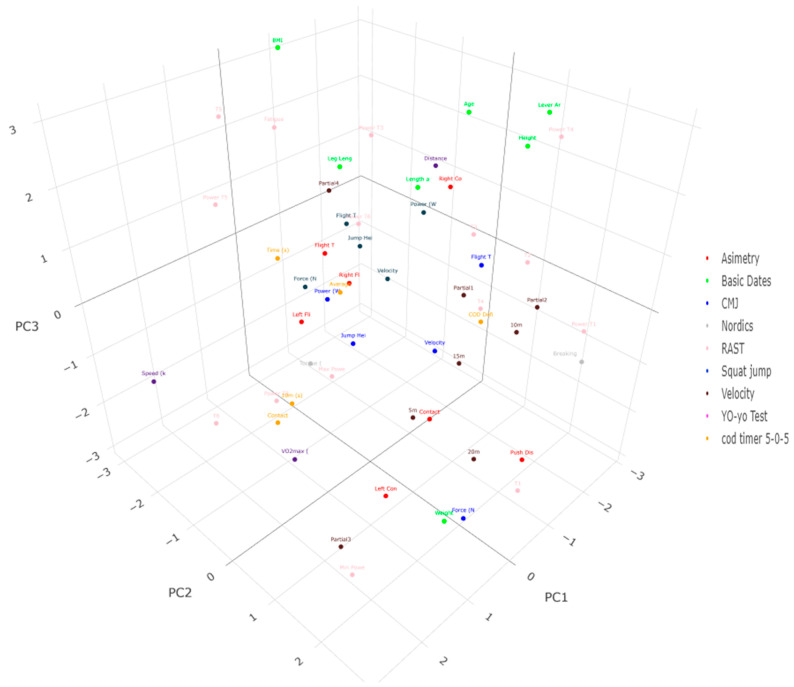
Distribution of the 3 rotated principal components on the fitness profile for attackers. Each color represents a group of variables.

**Figure 3:**
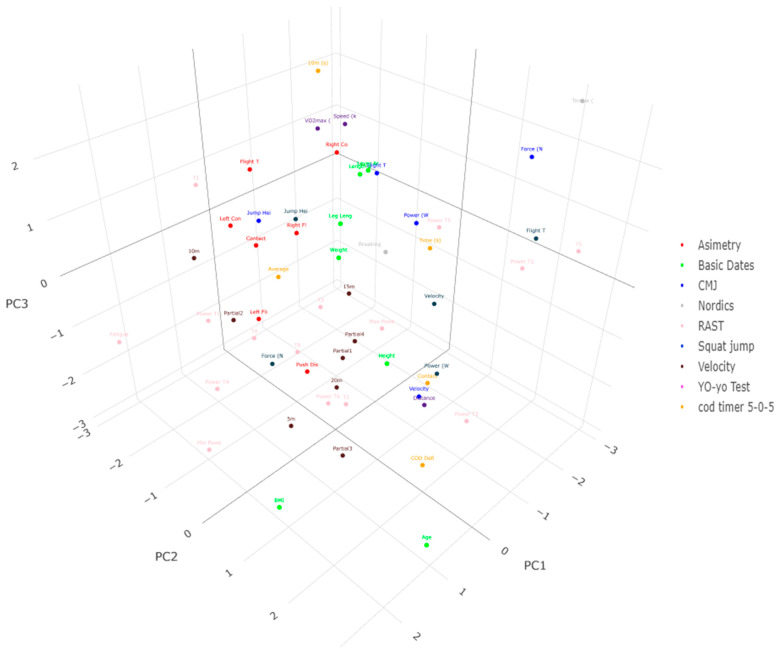
Distribution of the 3 rotated principal components on the fitness profile for defenders. Each color represents a group of variables.

The principal components analysis (PCA) shows the analysis for each of the physical fitness tests in the evaluated players in Table 5 and Figure 2 and Figure 3. Three PCs were extracted from the young soccer players according to their role, representing 74.96% and 96.26% of the total variance for attackers and defenders, respectively. The PC1 for attackers considered variables of strength, asymmetry, change of direction, and power. PC2 only considers strength and power variables. PC3 considered variables of strength, speed, endurance, and power. For defenders, PC1 considered strength, asymmetry, and power. PC2 analyzed variables of strength, asymmetry, change of direction, and power. Finally, PC3 only grouped speed variables.

The PC1 for attackers represents 22.62% of the total variance and is composed of Flight Time (ms) and Force (N) for the CMJ, Flight Time (ms) and Power (W) for the SJ, Contact Time Asymmetry (%), Right Contact Time (ms), and Right Flight Time (ms) for the SLCMJ, Average Speed (km/h) in the COD-Timer 5-0-5 test, and the minimum power from the RAST. The PC2 for attackers represents 74.96% of the total variance and is characterized by Torque (Nm) and Breaking Angle (A°) in the Nordic Hamstring, as well as Power T5, T6, and Power T6. The PC3 represents 56.27% of the total variance and is characterized by Power (W) in the CMJ, Torque (Nm) in the Nordic Hamstring, Speed at 5 m, Vo2max (mL-kg-min), T3, and T5 in the RAST. The PCA for defenders revealed that PC1 was highlighted by Force (N), Velocity (ms), and Power (W) for the SJ, Push Distance, Contact Time Asymmetry (%), Flight Time Asymmetry (%), Left Contact Time (ms), and Left and Right Flight Time (ms) in the SLCMJ test. This PC accounted for 23.37%. Meanwhile, PC2 represented 96.26% of the total variance and was characterized by Force (N), Velocity (ms), Push Distance in SLCMJ, and Breaking Angle (A°) in Nordic Hamstring, Contact Time in the COD-Timer 5-0-5 test, and, finally, high values in Power T5, T6, and Power T6 in the RAST. The final PC3 was notable only in the 5 m speed test.

## 4. Discussion

This study aimed to characterize and identify the level of physical fitness and profiles of youth soccer players about their roles on the field. A total of 36 players were evaluated through various physical fitness tests. The main findings of this study included significant associations between strength capacities measured through the SJ, CMJ, and SLCMJ tests, the ability to repeat sprints, and actions at high intensity, as well as higher speeds achieved in 5 and 10 m linear sprints and the COD test for attackers. By contrast, defenders excelled in tests that measured aerobic power and Vo2max, as well as Nordic Hamstring and linear speed over 15 m.

Previous studies have analyzed factors of physical fitness, particularly balance, strength, reaction time, and speed in both adult amateur and professional players [80], as well as the specificity of jumping, acceleration, and change of direction in adult players (23.2 ± 2.36 years) [81]. However, there are limited studies in the literature regarding the study of physical fitness in young soccer players using PCA. This study represents one of the first attempts to understand the relationship between physical fitness profiles according to the roles in which players predominantly engage (attackers and defenders).

Soccer is a sport that encompasses multiple skills, where various physical fitness variables play a crucial role [58]. Among these, jumping performance and sprinting [82], power [83], strength, aerobic power, change of direction [84], and agility [85] stand out. In this regard, it has been confirmed that the young players in this study showed varying levels of strength, power, and speed, indicating that physical fitness performance is not the same for the different roles of players (attackers and defenders).

Moreover, other studies have also corroborated that beyond the type and sequence of training developed for young soccer players, neuromuscular capacity significantly enhances performance in 10 m sprints and agility [86]. However, it is important to note that various methodological options currently exist to stimulate training activity and exercise intensity, promoting specific adaptations to the demands of youth soccer [87].

In this context, the study conducted by Bujnovky et al. [21] determined that there is a significant influence on linear sprint performance (*p* < 0.01, np^2^ = 0.23), with midfielders (2.44 ± 0.08 s) and wing midfielders (2.47 ± 0.13 s), who fulfill both attacking and defensive roles, achieving higher performance levels in the 505 agility test compared to goalkeepers (2.61 ± 0.23 s). When comparing these results with those of this study, which did not differentiate positions into goalkeepers, defenders, wing defenders, midfielders, wing midfielders, wingers, and forwards but instead grouped them by the primary role they performed based on their positioning on the field, it was evident that in the COD 5-0-5 test, attackers exhibited better performance (2.61 ± 0.08 s) compared to defenders (2.69 ± 0.05 s).

Meanwhile, other key performance variables, such as the YYIR1 and RSA tests, showed that field players also exhibited superior performance compared to goalkeepers (*p* < 0.01) [15]. For this study, the defenders showed better performance in the distance covered (1865.45 ± 306.97 m) and a higher Vo2max value (52.07 ± 2.58 mL/min/kg) in the YYIR1 test, while in the RAST, they exhibited a lower percentage of fatigue (34.8 ± 4.48%), which may be associated with a greater number of moderate-intensity actions of longer duration. By contrast, attackers were characterized by expressing a higher maximum power (295.89 ± 45.43 W) compared to defenders (228.75 ± 29.72 W).

Coaches tend to primarily consider technical [88] and tactical characteristics when determining a player’s position during competition [89,90], as well as adapting collective play to individual profiles to optimize performance [32]. However, physical fitness performance in young soccer players has also been reported in several studies as one of the key characteristics for adapting to the specific demands of the sport, combining agility, speed, strength, and aerobic–anaerobic processes [91,92,93,94].

Similarly, studies using multivariate methods have been conducted on young soccer players, revealing that physical capabilities can help explain sports performance [58] in the Colombian context. By contrast, in Chinese soccer, players classified by coaches as talented were characterized by higher expression in the development of physical skills compared to those deemed less talented [95]. In response to this, understanding the relationships between physical capabilities can assist coaches in managing specific programs that address not only the demands of the sport but also the needs of each context.

Previous studies have employed PCA methodology to define athlete profiles, recognizing specificities across various sports such as ice hockey [96], basketball [97], rugby [98,99], and team sports [100]. Additionally, studies have been developed using PCA in soccer players. In this regard, the systematic review conducted by Rojas-Valverde et al. [101] on the use of PCA for analyzing team sports reveals that soccer is the sport with the largest number of studies (n = 16), explaining 77.1% of the total variance. However, while there is a moderate amount of research using PCA to establish different relationships, existing studies have primarily focused on analyzing physiological variables, such as the isokinetic strength ratio between hamstrings and quadriceps [102] and metabolic markers of salivary fatigue in consecutive matches [103], as well as evaluating performance in professional players during competitions [65,104,105] and the effects of small-sided games on technical and tactical variables [106,107].

Similarly, PCA has been employed in studies to analyze gameplay position and outcomes in professional players, determining that winning teams exhibited different profiles, characterized mainly by their ability to recover the ball and organize offensive plays using penetrating passes to increase the number of shots on goal [108]. These results can be associated with the primary characteristics of defenders and attackers identified in this study, where defenders were defined by high levels of aerobic power and speed over 15 m, which they can use to fulfill the role of ball recovery, while attackers achieved better levels in the 5 and 10 m sprints, as well as in explosive actions that may be related to rapid offensive organization in pursuit of a goal.

For this purpose, PCA emerges as a statistical method that allows for the description of a data set in terms of new uncorrelated variables (‘components’), seeking to explain the variables that may be most determinant in player performance [97]. Mathematical methods are being applied to reduce the dimensions that explain physical performance in soccer, relating the nomination scale for identifying soccer talent and physical fitness [109], as well as the design of training, performance analysis, and talent identification based on principal component analysis [110].

From this analysis, the study developed by Castro-Infantes et al. [109] found that the two principal components represented 73% of the total variance, characterized by capabilities such as strength, speed, and agility. In this study, there are two principal components, one for attackers and another for defenders, representing 74.96% and 71.69% of the total variance, respectively, characterized in the evaluated sample by capabilities such as strength, SLCMJ (Single-Leg Countermovement Jump), and power. Additionally, the PCA revealed that attackers are characterized by a manifestation of high-intensity actions in which explosive strength and anaerobic power stand out, while defenders are characterized by actions dependent on endurance. Our findings indicate that specific training programs must be developed not only in response to the position played but also to the role performed and the particularities of each context. Thus, this information derived from the PCA could be used by all stakeholders to verify the impact of each training program on the adaptive processes of each athlete [111,112].

### Limitations and Future Prospects

This research represents a first attempt to identify physical profiles in response to the role played by young soccer players based on principal component analysis, although several limitations should be noted. The first limitation is related to the sample size and specific competitive level, which makes the data particular to the study population, complicating generalization. Another difficulty is associated with the division of players by roles, such as defenders (goalkeepers, center-backs, full-backs, and midfielders) and attackers (forwards, wingers, and strikers), which may pose another challenge in interpreting the results, as all players in soccer fulfill both attacking and defensive roles.

Considerations of profiles based on playing roles can help facilitate processes for identifying and evaluating performance in young players in response to age and competitive level. This, in turn, also opens the door for future research to consider performance evaluation based on profiles about principal components throughout an entire season, as well as comparisons between microcycles and in response to training or competition demands.

Finally, it is necessary to consider that the PCA approach and the sensitivity of small sample sizes may not generalize findings in response to other sporting contexts where attempts are made to replicate this type of statistical technique.

## 5. Conclusions

Three principal components were identified for each playing role (attack and defense), revealing that PC1 and PC3 for attackers were significant in the CMJ performance. PC1 for both attackers and defenders was characterized by high levels in the SLCMJ variables. PC2 for attackers was prominent in Nordic Hamstring strength and in power expressed in the RAST. PC3 for both attackers and defenders stood out in 5 m speed and Vo2max. PC2 for defenders was characterized by high levels of explosive strength in the SJ and contact time in the COD-Timer 5-0-5.

In youth soccer, there are playing positions that fulfill both attacking and defensive roles, so the differences in certain physical fitness variables among players highlight the need to develop training proposals tailored to the specificity of the playing position, in line with the systems of play employed and the predominance of the role that players occupy, both in defense and attack. Based on this, soccer professionals should consider these results to contextualize the importance of measuring physical fitness variables related to strength, sprint repetition capacity, high-intensity actions, aerobic power, speed, and change of direction in areas responsible for identifying and developing talent.

### Practical Applications

Based on the findings of this study, different practical applications can be proposed concerning the various physical fitness capabilities in young soccer players based on principal component analysis that can be considered by coaches and trainers for player training. These are as follows:

▪ Since attackers displayed higher physical fitness values, particularly in-game actions that rely on explosive power, accelerations, decelerations, and anaerobic speed, it would be relevant to individualize the training workload according to the role they occupy on the field.
▪ Defenders demonstrated high levels in the Nordic Hamstring test and also covered greater distances with a higher VO2max. This may enable them to perform actions where aerobic power is critical. This information is useful, as it would allow for the design of training tasks that respond to the specificity and individuality of the players.
▪ Understanding the physical fitness profile of players based on their roles on the field would enable coaches and physical trainers to design game systems related to the capabilities exhibited by the players, where attackers perform better in short-distance actions (5 and 10 m) and defenders excel at 15 m due to the movements they commonly make.

## Figures and Tables

**Table 1:** Basic data of the evaluated sample.

Variable	Defenders (n = 15)	Attackers (n = 21)
Age (years)	14.23 ± 0.18	14.07 ± 0.13
Weight (kg)	43.54 ± 2.77	48.33 ± 4.13
Height (cm)	1.56 ± 0.04	1.6 ± 0.04
BMI (kg/m^2^)	17.66 ± 0.68	18.87 ± 1.24
Leg Length (cm)	95.82 ± 2.56	97.25 ± 3.13
Length at 90 (cm)	65.93 ± 3.14	64.07 ± 2.88
Lever Arm (cm)	114.3 ± 0.03	117.6 ± 0.03

**Note**: kg: kilograms; cm: centimeters; BMI: body mass index.

**Table 2:** Descriptive and inferential analysis of differences related to strength variables of young soccer players between defenders and attackers.

Test	Measured Variable	Defenders	Attackers	W	*p*	r	R-b
CMJ	Jump Height (cm)	26.74 ± 1.76	28.69 ± 2.88	−1.2	0.24	−0.33	Small effect
CMJ	Flight Time (ms)	465.62 ± 15.2	481.16 ± 26	−1.08	0.29	−0.33	Small effect
CMJ	Force (N)	823.05 ± 81.18	861.38 ± 85.58	138	0.42	0.33	Small effect
CMJ	Velocity (m/s)	1.14 ± 0.04	1.18 ± 0.06	−1.12	0.27	−0.33	Small effect
CMJ	Power (W)	947.98 ± 112.95	1044.28 ± 122.68	121	0.18	0.33	Small effect
SJ	Jump Height (cm)	24.14 ± 1.79	26.85 ± 1.92	−1.97	0.06	−0.33	Small effect
SJ	Flight Time (ms)	442.05 ± 16.25	468.5 ± 15.22	−2.22	0.03 *	−0.33	Small effect
SJ	Force (N)	787.46 ± 81.98	847.53 ± 73.9	117	0.14	0.33	Small effect
SJ	Velocity (m/s)	1.08 ± 0.04	1.14 ± 0.04	−1.99	0.05	−0.33	Small effect
SJ	Power (W)	863.9 ± 112.63	969.59 ± 104.09	108	0.08	0.33	Small effect
SLCMJ Asy	Push Distance (hp0, in m)	0.3 ± 0.02	0.33 ± 0.02	−1.58	0.12	0	Very small
SLCMJ Asy	Contact Time (%)	7.15 ± 2.62	13.12 ± 4.55	97	0.04 *	−0.33	Small effect
SLCMJ Asy	Flight Time (%)	14.61 ± 3.89	7.02 ± 2.68	2.85	0.01 *	0.33	Small effect
SLCMJ Asy	Left Contact Time (ms)	300.89 ± 56.39	342.93 ± 42.88	161.5	0.93	−0.33	Small effect
SLCMJ Asy	Right Contact Time (ms)	300.2 ± 56.24	350.87 ± 29.54	137	0.39	0.33	Small effect
SLCMJ Asy	Left Flight Time (ms)	274.48 ± 51.11	271.67 ± 32.71	205.5	0.22	0.33	Small effect
SLCMJ Asy	Right Flight Time (ms)	262.43 ± 35.73	270.13 ± 27.09	158	0.84	−0.33	Small effect
Nor Ham	Torque (Nm)	212.09 ± 23.12	204.11 ± 24.81	0.45	0.66	−0.33	Small effect
Nor Ham	Breaking Angle (A°)	122.69 ± 3.5	115.27 ± 5.36	2.38	0.02 *	0.33	Small effect

**Note**: *p*: significance; r: effect size; R-b: Rank-biserial; cm: centimeters; N: newton; ms: milliseconds; s: seconds; W: watts; Nm: Newton-meter; CMJ: Countermovement Jump; SJ: Squat Jump; SLCMJ Asy: Single-Leg Countermovement Jump Asymmetry; Nor Ham: Nordic Hamstring. * *p* < 0.05.

**Table 3:** Descriptive and inferential analysis of the differences related to the speed and change of direction variables in youth soccer players between defenders and attackers.

Test	Measured Variable	Defenders	Attackers	W	*p*	r	Rank-Biserial
Speed	5 m	1.07 ± 0.02	1.05 ± 0.04	1.06	0.3	0.4	Small effect
Speed	10 m	1.94 ± 0.05	1.93 ± 0.07	0.2	0.85	0.33	Small effect
Speed	15 m	2.71 ± 0.06	2.72 ± 0.1	−0.11	0.92	−0.33	Small effect
Speed	20 m	3.44 ± 0.08	3.44 ± 0.14	0.04	0.97	0.33	Small effect
COD-Timer 5-0-5	Time (s)	2.69 ± 0.05	2.61 ± 0.08	246.5	0.01 *	0.33	Small effect
COD-Timer 5-0-5	Average Speed (km/h)	7.77 ± 0.13	7.99 ± 0.23	−1.71	0.1	−0.33	Small effect
COD-Timer 5-0-5	Contact Time (ms)	367.6 ± 39.28	386.75 ± 34.39	−0.68	0.5	0.33	Small effect
COD-Timer 5-0-5	10 m (s)	1.95 ± 0.04	1.91 ± 0.08	0.8	0.43	0.33	Small effect
COD-Timer 5-0-5	COD Deficit (s)	0.75 ± 0.05	0.7 ± 0.08	207	0.2	−0.33	Small effect

**Note**: *p*: significance; r: effect size; m: meters; s: seconds; km: kilometers; h: hours; ms: milliseconds. * *p* < 0.05.

**Table 4:** Descriptive and inferential analysis of the differences related to aerobic power and running-based anaerobic speed variables among youth soccer players between defenders and attackers.

Test	Measured Variable	Defenders	Attackers	W	*p*	r	Rank-Biserial
YYIR1	Distance (m)	1865.45 ± 306.97	1672 ± 426.69	0.74	0.46	−0.33	Small effect
YYIR1	Vo2max (mL/min/kg)	52.07 ± 2.58	50.44 ± 3.58	0.74	0.46	−0.33	Small effect
RAST	T1	6.28 ± 0.18	5.92 ± 0.22	244.5	0.01 *	0.39	Small effect
RAST	Power T1	224.12 ± 30.31	295.53 ± 45.47	79	0.01 *	−0.33	Small effect
RAST	T2	6.63 ± 0.16	6.31 ± 0.24	2.31	0.03 *	0.33	Small effect
RAST	Power T2	187.68 ± 22.07	243.49 ± 34.8	83	0.01 *	−0.33	Small effect
RAST	T3	6.72 ± 0.18	6.56 ± 0.32	0.92	0.36	−0.33	Small effect
RAST	Power T3	182.85 ± 24.8	222.07 ± 41.2	120	0.17	0.33	Small effect
RAST	T4	7.02 ± 0.24	6.73 ± 0.24	1.61	0.12	−0.33	Small effect
RAST	Power T4	162.4 ± 23.71	198.79 ± 26.7	−1.97	0.06	0.33	Small effect
RAST	T5	7.00 ± 0.18	6.89 ± 0.33	0.66	0.51	−0.33	Small effect
RAST	Power T5	160.61 ± 20.29	188.21 ± 29.53	−1.56	0.13	−0.33	Small effect
RAST	T6	6.93 ± 0.18	6.77 ± 0.25	1.04	0.3	0.33	Small effect
RAST	Power T6	164.3 ± 18.8	193.92 ± 22.9	−1.96	0.06	−0.33	Small effect
RAST	Max Power	228.75 ± 29.72	295.89 ± 45.43	84	0.01 *	−0.33	Small effect
RAST	Min Power	147.81 ± 19.13	173.93 ± 23.37	106	0.07	−0.33	Small effect
RAST	Fatigue Index (%)	34.8 ± 4.48	40.28 ± 3.63	−1.73	0.09	0.33	Small effect

**Note**: *p*: significance; r: effect size; m: meters; mL: milliliters; min: minutes; kg: kilograms; T: time; max: maximum; min: minimum. YYIR1: Yo-Yo Intermittent Recovery Test Level 1; RAST: Running-based anaerobic sprint test; * *p* < 0.05.

**Table 5:** Principal component analysis by game role with their respective variances and % variance explained.

	PC	PC1	PC2	PC3	PC1	PC2	PC3
Test	Measured Variable	Attacker			Defender		
CMJ	Jump Height (cm)						
CMJ	Flight Time (ms)	0.17					
CMJ	Force (N)	0.17					
CMJ	Velocity (m/s)						
CMJ	Power (W)			0.15			
SJ	Jump Height (cm)						
SJ	Flight Time (ms)	0.17					
SJ	Force (N)				0.15	0.16	
SJ	Velocity (m/s)				0.15	0.17	
SJ	Power (W)	0.17			0.18		
SLCMJ Asy	Push Distance (hp0, in m)				0.15	0.17	
SLCMJ Asy	Contact Time Asymmetry (%)	0.17			0.18		
SLCMJ Asy	Flight Time Asymmetry (%)				0.15		
SLCMJ Asy	Left Contact Time (ms)				0.15		
SLCMJ Asy	Right Contact Time (ms)	0.17					
SLCMJ Asy	Left Flight Time (ms)				0.15		
SLCMJ Asy	Right Flight Time (ms)	0.17			0.18		
Nordic Hamstring	Torque (Nm)		0.15	0.19			
Nordic Hamstring	Breaking Angle (A°)		0.18			0.16	
Speed	5 m			0.25			0.28
Speed	10 m						
Speed	15 m						
Speed	20 m						
COD-Timer 5-0-5	Time (s)						
COD-Timer 5-0-5	Average Speed (km/h)	0.16					
COD-Timer 5-0-5	Contact Time (ms)					0.15	
COD-Timer 5-0-5	10 m (s)						
COD-Timer 5-0-5	COD Deficit (s)						
YYIR1	Distance (m)						
YYIR1	Vo2max (mL/min/kg)			0.15			
RAST	T1						
RAST	Power T1						
RAST	T2						
RAST	Power T2						
RAST	T3			0.20			
RAST	Power T3				0.16		
RAST	T4						
RAST	Power T4						
RAST	T5			0.25			
RAST	Power T5		0.31			0.20	
RAST	T6		0.30			0.20	
RAST	Power T6		0.30			0.20	
RAST	Max Power						
RAST	Min Power	0.19			0.17		
RAST	Fatigue Index (%)						
	Eigenvalue	2.26	7.49	5.62	2.33	9.62	5.94
	Variance	0.396	0.131	0.098	0.410	0.168	0.104
	%Variance	22.62	74.96	56.27	23.72	71.69	52.67

**Note.** PC: principal components; m: meters; mL: miniliters; min: minutes; kg: kilograms; YYIR1: Yo-Yo Intermittent Recovery Test Level 1; RAST: Running-based anaerobic sprint test; T: time; max: maxime; min: minimum; cm: centimeters; N: newton; ms: milliseconds; s: seconds; W: watts; Nm: Newton-meter.

## Data Availability

The data presented in this study are available on request from the corresponding author.
